# New Method for Simultaneous Arsenic and Selenium Speciation Analysis in Seafood and Onion Samples

**DOI:** 10.3390/molecules26206223

**Published:** 2021-10-15

**Authors:** Katarzyna Karaś, Anetta Zioła-Frankowska, Marcin Frankowski

**Affiliations:** 1 Department of Analytical and Environmental Chemistry, Faculty of Chemistry, Adam Mickiewicz University in Poznan, 61-614 Poznan, Poland; katarzyna.karas@amu.edu.pl; 2 Department of Analytical Chemistry, Faculty of Chemistry, Adam Mickiewicz University in Poznan, 61-614 Poznan, Poland; anettazf@amu.edu.pl

**Keywords:** simultaneous speciation analysis, arsenic, selenium, seafood samples, onion samples, LC–ICP–MS

## Abstract

This paper presents a new method for the simultaneous speciation analysis of arsenic (As(III)-arsenite, As(V)-arsenate, DMA-dimethylarsinic acid, MMA-methylarsonic acid, and AsB-arsenobetaine) and selenium (Se(IV)-selenite, Se(VI)-selenate, Se-Methionine, and Se-Cystine), which was applied to a variety of seafood and onion samples. The determination of the forms of arsenic and selenium was undertaken using the High-Performance Liquid Chromatography Inductively Coupled Plasma Mass Spectrometry (HPLC–ICP–MS) analytical technique. The separation of both organic and inorganic forms of arsenic and selenium was performed using two analytical columns: an anion exchange column, Dionex IonPac AS22, containing an alkanol quaternary ammonium ion, and a double bed cation–anion exchange guard column, Dionex Ion Pac CG5A, containing, as a first layer, fully sulfonated latex for cation exchange and a fully aminated layer for anion exchange as the second layer. The ammonium nitrate, at pH = 9.0, was used as a mobile phase. The method presented here allowed us to separate the As and Se species within 10 min with a suitable resolution. The applicability was presented with different sample matrix types: seafood and onion.

## 1. Introduction

Seafood is an important source of protein, polyunsaturated fatty acids such as omega-3 and omega-6, phospholipids, B vitamins, the fat-soluble vitamins A and D, phosphorus, potassium, sodium, calcium, magnesium, iron, iodine, and selenium [1,2,3]. Seafood is considered to be the largest reserve of polyunsaturated fatty acids, especially omega-3 fatty acids [4], which are not synthesized in the human body and can only be obtained from food [5]. However, despite the recognized benefits, fish and other marine products may pose a risk to human health through the accumulation of pollutants from the marine environment and biomagnification along the food chain [4]. They are recognized by toxicologists as the main source of toxic substances, including trace metals and persistent organic pollutants [6], such as dioxins, furans, and polychlorinated biphenyls [7].

Metals and semimetals, from natural and anthropogenic sources, can end up in the water, where they pose serious threats because they are toxic and long-lasting and are subject to bioaccumulation and biomagnification in the food chain [8]. All aquatic invertebrates accumulate trace metals from food, suspended matter, and directly from seawater, even when they do not need them. Some may even take up certain metals to levels higher than those in the surrounding waters, so the same concentration in one species may be considered low and another too high [9]. Human organs, such as the liver, kidneys, central nervous system, mucosa, gastrointestinal tract, and reproductive system, can be seriously damaged by the consumption of products contaminated with dangerous trace metals [2]. Toxicity and the resulting health risks depend on the concentration, but chronic exposure to relatively low concentrations of arsenic, cadmium, mercury, and lead can cause health problems [10]. Toxicity is also influenced by the interaction of elements or their forms with each other, and the total toxicity depends on the pH, salinity, and amount and type of dissolved organic matter [11].

According to European Union regulations, the maximum dose of inorganic arsenic in rice and rice products should not exceed 0.3 mg·kg^−1^ fresh mass and should not exceed 0.5 mg·kg^−1^ in aquatic animals and their products [12,13,14]. An adequate daily intake of selenium ranges from 15 to 70 µg a day and depends on age [15].

However, the content of a given element is not enough to determine its toxicity. Different forms of the same element may have different physical, chemical, and toxic properties [16]. For example, the inorganic form of arsenic is more toxic than the organic [1]. Therefore, to define toxicity, biotransformation, and the impact on living organisms of a given element, it is important to research its chemical speciation [17,18].

Marine products are one of the largest sources of arsenic in the human diet, and over 50 different forms and compounds of this element have been identified [19,20,21]. They may contain compounds such as arsenics and arsenates, salts containing arsenic (III) and arsenic(V), MMA, DMA, AsB, and arsenic sugars [21]. Marine organisms, including fish, crustaceans, and seaweed, are high in total arsenic, but more than 90% is in organic form [22].

The inorganic forms of arsenic are more toxic than the organic forms, with arsenic in the third oxidation state being more toxic than in the fifth [23]. MMA and DMA are less toxic than their metabolites, which contain arsenic (III). Arsenobetaine, arsenocholine, and arsenosugars are relatively nontoxic [24]. Toxicity decreases with increasing methylation, so MMA is more toxic than DMA [23]. MMA and DMA are more toxic than arsenosugars but less bioavailable [25]. Inorganic forms of selenium, such as selenite and selenate, show high toxicity in high doses. On the other hand, selenocystine, Se-methylselenocysteine, and selenomethionine, which are organic forms of selenium, have many health benefits [26,27]. Marine products, including fish and mussels, bioaccumulate selenium and are therefore an ideal source of this micronutrient [28]. On the other hand, vegetables that have a relatively low selenium content, such as onion and garlic, are able to accumulate selenium in high content. In addition, it was suggested that selenium has anticarcinogenic properties. Considering that various species of Allium plants, including onions, are Se accumulators, it is worth finding out about the species of this element.

The aim of this research was to (1) develop a method for the simultaneous speciation analysis of arsenic (As(III)-arsenite, As(V)-arsenate, DMA-dimethylarsinic acid, MMA-methylarsonic acid, and AsB-arsenobetaine), antimony (Sb(III), Sb(V)), chromium (Cr(III), Cr(VI)), and selenium (Se(IV)-selenite, Se(VI)-selenate, Se-Methionine, and Se-Cystine) during one analysis by hyphenated technique High-Performance Liquid Chromatography Inductively Coupled Plasma Mass Spectrometry (HPLC–ICP–MS); and (2) apply the new method to speciation analysis of seafood and onions matrices. The selected samples are widely consumed all over the world, and there is not currently a single and accurate method for simultaneous As, Sb, Cr, and Se speciation in all these matrices.

## 2. Results and Discussion

### 2.1. Simultaneous Arsenic and Selenium Speciation Analysis

**Figure 1:**
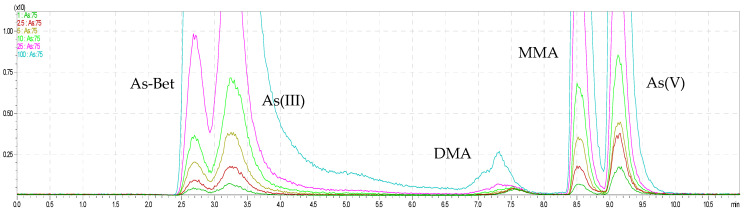
The overlapped chromatograms of As species for HPLC–ICP–MS.

**Figure 2:**
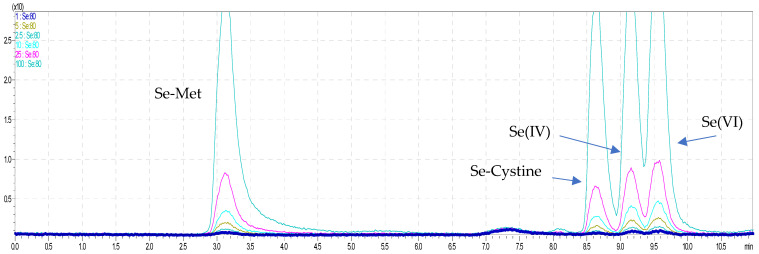
The overlapped chromatograms of Se species for HPLC–ICP–MS.

The As and Se species were completely resolved on use in the Dionex IonPac AS22 analytical column connected to the Dionex IonPac CG5A guard column with gradient elution. We applied the gradient elution to improve the separation of As and Se species. A similar way to achieve full separation of arsenicals was presented in a study by Kohlmeyer et al. (2002) [29], where the method with anion exchange with nitric acid eluents together with a doubly charged ion-pairing agent was applied to separate anions and cations of arsenicals together in a single chromatographic run. Additionally, the Sloth et al. (2003) [30] used the gradient elution for separation the organoarsenicals in one chromatographic run using cation exchange. The goal of present study was to make the simultaneous speciation of arsenic and selenium in one run; that is why we used anion exchange column in combination with an anion–cation exchange guard column, which was not yet applied in speciation analysis of metalloids. The most commonly column for speciation of arsenic is anion exchange chromatography with Hamilton PRP-X100 column [31], which we tested but did not obtain full separation of arsenic and selenium forms (Table 1). Furthermore, Sakai et al. (2001) [32] also used anion and cation exchange columns to separate eight arsenicals. In this study, we applied the combination of anion and cation exchange columns for speciation analysis of arsenicals in seafood. It is well known that chromatographic approaches, such as ion-pairing reversed-phase, ion-exchange, ion exclusion, and reversed-phase chromatographies, are reported to facilitate speciation of arsenicals in marine sample extracts. Moreover, it was confirmed that methylated arsenicals and AsSugars were successfully isolated on anion exchange columns, while cation exchange columns were efficient for separation for AsB, AsC, DMA, TMAO, TETRA, and DMAA, while for AsLipids, RP–LC was typically used with a C8 or C18 column [33]. In addition, in the literature was found that arsenobetaine (AsB) at pKa = 2.18 is zwitterionic; that is why, in our case, the use of a bifunctional column was justified [33]. The chromatographic separation obtained for the analytes in standard solutions at the mentioned concentrations are presented in Figure 1 and Figure 2. It is worth noticing that, in the case in speciation analysis of arsenic, the use of separate chromatographic methods is recommended for different groups of arsenicals because it is difficult to achieve efficient separation of anionic and cationic species using a single method. For example, in the study by Wolle et al. (2021) [34] the As(III), As(V), DMA, and MMA were separated by the anion exchange method, and AsB was separated on a cation exchange column in aqueous extracts analyzed by HPLC−ICP–MS, but each column was used separately. Morevoer, the AsB form was separated in 20 min (cation exchange) and As(V) form in 30 min (anion exchange) by HPLC–ICP–MS. Similar to our study, Sakai et al. (2001) used anion and cation exchange columns to separate eight arsenicals [32]. They obtained good resolution, but the serious limitation to apply this method was long chromatographic run time of 40 min. Furthermore, in the case of applied ion exclusion chromatography with ICP–MS detection by Nakazato et al. (2000) for separation of arsenic species, the long chromatographic run times (up to 60 min) was the main disadvantage of this method [35]. In this study, we obtained in one run of not only arsenic species but also selenium species in short chromatographic run time (10 min) with a sufficient resolution, which is to opposite to previous studies, where species of these metals were analyzed separately [36,37,38,39]. Moreover, the additional advantage of this research was the successful separation of the rarely presented organic form of selenium, Se-cystine. Other studies have focused only on total Se content [40].

Another case of this study was connected with the statement from review by Francesconi and Kuehenlt (2004), where the authors wrote that during chromatographic process can occur possibly effecting changes in arsenic species. Regarding this, they recommended to include in the research a statement about the recovery of arsenic from chromatographic columns [31]. For this reason, in this study, the column (chromatographic) recovery was calculated as the ratio of the sum of the concentrations of the chromatographed species and the concentration od As and Se in the corresponding aqueous extract, analyzed by ICP–MS. We obtained the chromatographic recoveries in the range 93–97% and 92–98% for As and for Se % (onions) and 93–98% for As and 90–97% for Se (seafood). The good recovery values confirmed that the conditions of chromatographic separation in this study were efficient, and mostly all analytes were fully separated and eluted on the column(s).

### 2.2. Speciation Analysis of Water-Soluble Arsenic and Selenium by HPLC–ICP–MS

**Figure 3:**
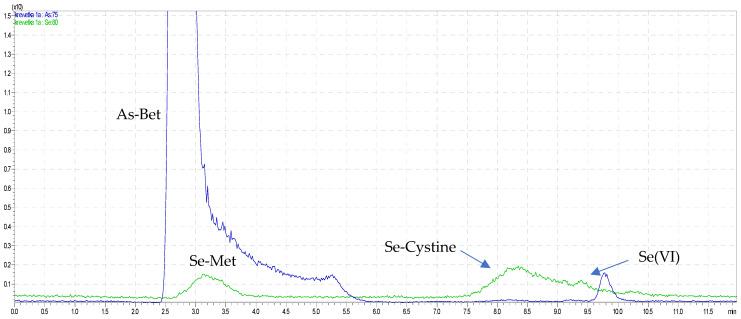
The overlapped chromatograms of As and Se species in red shrimp.

**Figure 4:**
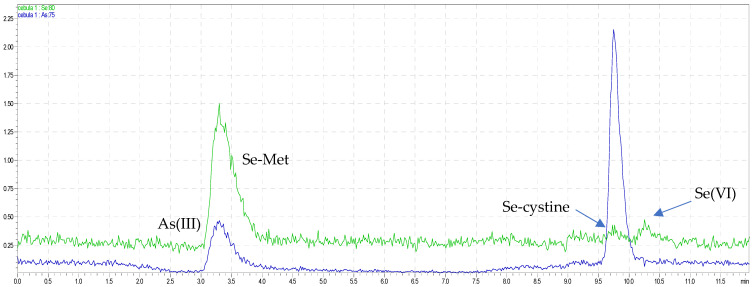
The overlapped chromatograms of As and Se species in green bio onions.

The applicability of the new method was successfully performed to simultaneous analysis of five arsenic and four selenium species, AsB, As(III), As(V), DMA, MMA, Se-cystine, Se-Met, Se(IV), and Se(VI), in onions and seafood samples. Sample chromatograms from the shrimp and onion analyses are presented in Figure 3 and Figure 4, and the quantitative results from the determination of inorganic and organic arsenic and selenium species are shown in tables (Table 1).

It is known that dietary seafood are considered to be the most important source of human exposure to As contamination. Moreover, the biotoxicity and bioavailability of As strongly depend on its chemical species, in the same way that forms of arsenite and arsenate are known to be the most toxic forms, which are categorized to a class 1, no threshold carcinogen, while organic species, for example, methylarsonic acid (MMA), dimethylarsinic acid (DMA), and arsenobetaine (AsB), are much less toxic to marine organisms and humans [19]. In this study for all seafood samples the inorganic arsenic concentration was below LOD. In contrast to inorganic forms, organic arsenic species were found in the seafood samples. The predominant form of the arsenic species was arsenobetaine in the range 2.221–20.69 mg·kg^−1^. The highest concentration was found in Argentine red shrimp, and the lowest, in white shrimp. In the case of speciation analysis of As in shellfish, similar to that in this study, the highest concentration was obtained of AsB forms. For shellfish, where the clam samples dominate, the range of AsB was 7.878–25.817 mg·kg^−1^, which was analyzed in extract after acid mineralization [19]. In this study, in the case of clams, AsB was determined at a similar concentration level, but it should be emphasized that the sample was extracted with water and not with acids. For this reason, obtained results are in accordance with the statement that AsB as a polar molecule can be easily extracted with aqueous solution [41] Moreover, water can be helpful to effectively extract hydrophilic arsenicals while keeping their chemical integrity [33]. On the other hand, the results for AsB in water-soluble extract of seaweeds were much lower (range 0.017–0.357 mg·kg^−1^), but it should be emphasized that, contrary to our research, toxic forms iAs (the highest was above 50 mg·kg^−1^) were determined in most algae, and in all seaweeds, the DMA form was determined in the range (0.049–2.924 mg·kg^−1^) [33], which was also higher compared to this study. Liu et al. (2020) [42] conducted the speciation analysis in edible tissues of mitten crabs and obtained that AsB was the predominant form among the six arsenic species (in the range 0.015–0.042 mg·kg^−1^), and the ranking was as follows: AsB > MMA > As(III) > AsC > DMA > As(V). For seafood, the order is as follows: AsB > MMA > DMA > As(III) = As(V), but with much higher concentration of the AsB form. Taking into consideration the toxicity of arsenic species, the categories based on the International Agency for Research on Cancer (IARC) classification of the carcinogenicity of arsenic species were suggested. First is toxic inorganic arsenic fraction; second, AsB, which is established as nontoxic; and the last, leftover arsenicals, which may contain arsenosugars and other nonwater-extractable, lipophilic compounds. Results obtained in this study are in accordance with the finding that seafood is considered safe, owing to the benign nature of AsB that predominates and the low levels of iAs [33].

In the case of onion, inorganic arsenic was quantified in all samples, where only the As(III) form was found at the level below 0.2 mg·kg^−1^. According to the Smith et al. (2008) [41] conducted arsenic speciation in vegetables using trifluoroacetic acid extraction procedure, obtained arsenite and arsenate forms. The As(III) was minor component in the roots of radish, lettuce, and chard with much higher concentrations compared to the present study, while As(V) was dominant in the mung bean roots (more than 15 mg·kg^−1^) [41]. Bergquist et al. (2014) found As(III) and As(V) in carrot, lettuce, and spinach after water extraction, but mainly arsenite form in the range 0.01–2.73 mg·kg^−1^ (one result for lettuce root at 89.3 mg·kg^−1^) [43]. In vegetables (broccoli and cucumber), studies undertaken by Hacketkal et al. (2021) were similar, but only iAs forms were found in the range 0.003–0.024 mg·kg^−1^, which was much lower compared to the concentration of iAs in onion samples [44]. Results of speciation analysis in onion samples are in accordance with As speciation studies with other plants including Pityrogramma calomelanos [45] and Pteris vittata [46], in which was also identified the predominance of As(III) in plant tissue. It can be connected with reduction of As(V) to As(III) as a plant tolerance mechanism, or it can be connected with uptake of As(III) from soil. Moreover, the process of reduction of As(V) to As(III) may limit the As(V) toxicity [41]. Due to the total content of As in onion, Pinter et al. (2017) determined average 91.2 mg·kg^−1^ for bulbs and 77.6 mg·kg^−1^ for leaves of onion [46]. When we recalculated the value for iAs concentration as a fraction of 70% of total. As content, we obtained much higher data compared to present and previous studies (iAs- 63.84 mg·kg^−1^ for bulbs and for leaves 54.32 mg·kg^−1^) [47]. 

The new method developed here allowed for the simultaneous determination arsenic and selenium forms. Inorganic selenium species were found in most of the samples, but the predominant determined form was Se(VI), especially for seafood, and the major organic form was Se-methionine, which was found in both onions and seafood samples. The highest content of this form was found in white sweet onion. The Se-cystine form was found only in seafood, with the highest content in red shrimp. 

In the present study, in the onion samples, both inorganic and organic forms were obtained. Wróbel et al. (2004) found that the extracts obtained from leaves grown in the presence of Se(IV) and Se(VI) obtained Se(VI), Se-cystine, Se-methionine, and Se-methylselenocysteine forms, while in the hot water extract from onion bulbs, similar to the findings in this study, Se-methionine and additional Se-methylselenocysteine were also observed [48]. 

Further research on selenium speciation in enriched-by-Se(IV) onion samples was conducted by [49]. It should be underlined that they performed only qualitative speciation analysis and obtained for HCl extract Se-methylselenocysteine and Se-methionine forms, while for enzymatic extract, inorganic selenium, Se-cystine, and Se-methionine (the highest signal) forms [49]. In addition, SeMet was the dominant form in enzymatic extracts of plant food such as: buckwheat, green pea, strawberries, rice, cabbage, and kale, which were treatment by Se(IV) [50]. These results are in accordance with the present speciation data for onion samples and can be a confirmation of the statement that SeMet form has been found to be more bioavailable and less toxic than inorganic selenium species [51]. Furthermore, the sum concentration of species of selenium in onion samples was higher compared to seafood data. It can be suggested that this difference may have been caused by the fertilization method used for cultivation due to deficiency of selenium in the soil. Kapolna and Fodor (2007) estimated that, when onions are enriched with Se species, the total selenium content increases significantly, especially in the bulbs, which may lead to higher Se contents in green onion samples [52].

It is well known that selenium is an essential micronutrient for humans, and seafood is one of the major selenium sources [40]. Moreover, its toxicity and bioavailability strongly depends on its chemical form and concentration [50].

In seafood water extracts in the present study, in contrast to data for onion samples, the Se(VI) was the predominant form. However, higher concentration was marked for Se-cystine and SeMet forms. In seafood samples, the Se(IV) was marked only in white blanched shrimp. Jagtap et al. (2016) determined the SeMet was found in fish muscle and liver tissues after enzymatic hydrolysis in the range 1.0–5.4 mg·kg^−1^, which is much higher compared to this study [53]. Lower concentration (from 0.12–4.0 mg·kg^−1^) of SeMet was obtained by Sele et al. (2018), who confirmed that SeMet was the major Se species in muscle of Atlantic salmon fed both basal diets and diets supplemented with selenised yeast [54]. These findings are in agreement with the earlier statement that SeMet is more readily available than inorganic Se species [54], which can be explained by higher data for SeMet compared to Se(VI) in the present study. Taking into account the sum of all forms of selenium in water extracts, the marked range 0.13–1.47 mg·kg^−1^ was at the same level as the obtained total content of selenium in fish and shellfish (range 0.12–1.27 mg·kg^−1^) [40]; we also found a selenium level of 0.567 mg kg^−1^ in tuna, 0.346 mg kg^−1^ in scads, and 0.279 mg kg^−1^ in swordfish and in the range from 0.470 to 0.950 mg kg^−1^ in fish and mollusks [25].

## 3. Materials and Methods

### 3.1. Reagents and Standards

Ultrapure water (>18.2 MΩ·cm) obtained from a Milli-Q Direct 8 purification unit (Millipore, Burlington, MA, USA, Merck), was used to prepare all the solutions. Arsenic salts (sodium meta-arsenite (As[III], Sigma–Aldrich, St. Louis, MI, USA), sodium hydroxy arsenate heptahydrate (As[V], Sigma–Aldrich, St. Louis, MI, USA), sodium cacodylate trihydrate (DMA, Sigma–Aldrich, St. Louis, MI, USA), sodium methylarsenate (MMA, Supelco, Bellefonte, PA, USA), arsenobetaine (Sigma–Aldrich, St. Louis, MI, USA)), antimony salts (potassium antimony tartrate hydrate (Sb[III], Sigma–Aldrich, St. Louis, MI, USA), potassium hexahydroxy antimonate(V) (Sb[V], Sigma–Aldrich, St. Louis, MI, USA)), chromium salts (potassium chromate (Cr[III] Sigma–Aldrich, St. Louis, MI, USA), ammonium dichromate (Cr[VI] Sigma–Aldrich, St. Louis, MI, USA)), and selenium salts (sodium selenite (Se[IV], Sigma–Aldrich), sodium selenate (Se[VI], Sigma–Aldrich, St. Louis, MI, USA), seleno-l-cystine (Sigma–Aldrich, St. Louis, MI, USA), and seleno-l-methionine (Sigma–Aldrich, St. Louis, MI, USA)) were used to prepare standard solutions. Ammonium nitrate (NH_4_NO_3_), ammonium carbonate ((NH_4_)_2_CO_3_), ammonium decarbonate (NH_4_HCO_3_) (Sigma–Aldrich, St. Louis, MI, USA), and methanol (Merck, USA) were used to prepare the mobile phases. Ammonia or nitric acid (Sigma–Aldrich, St. Louis, MI, USA) was used to adjust the pH of the mobile phases. To avoid contamination, all glassware and storage bottles were kept in 10% (*v*/*v*) nitric acid for at least 48 h, rinsed three times with ultrapure water, and kept dry until use. 

### 3.2. Analytical Instruments

An ICPMS-2030 mass spectrometer (Shimadzu, Tokyo, Japan), directly coupled with a Prominence LC 20Ai inert system, was used for creating the new speciation method. The LC inert system allowed us to eliminate the possibility of metal background leaching from the mobile phase and enabled us to obtain the lowest possible detection limit, which is required for speciation analysis. The ICP–MS operated at 1.2 kW with 8.0 L·min^−1^ Ar plasma gas flow, 0.7 L·min^−1^ nebulizer Ar gas flow, and 1.1 L·min^−1^ auxiliary Ar gas flow. The concentric (MicroMist) nebulizer with 0.6 mL·min^−1^ (carrier) argon gas flow was used for nebulizing the HPLC eluate. The sampling depth was set to 5.0 mm. The spray chamber temperature was set to 3 °C. Optimized conditions of the collision cell were −90 V of cell gas voltage, 7.0 V of energy filter voltage, and a 9.0 mL·min^−1^ cell gas (He) flow rate. The HPLC was equipped with binary pumps LC 20Ai, an autosampler (SIL 20AC), a vacuum degasser (DGU20A3R), a heated column compartment (CTO 20AC), and a controller (CBM 20A) (Shimadzu, Japan). Polypropylene autosampler vials were used, because of the possibility of contamination of the samples with arsenate if using glass vials. The vials were cleaned with dilute nitric acid and thoroughly rinsed with ultrapure deionized water (UPW) (Merck, Kenilworth, NJ, USA) [55].

### 3.3. Method Development for Simultaneous Speciation Analysis

#### Analytical Column and Mobile Phase Effect

Based on the type of analysis and separate arsenic, antimony, chromium, and selenium forms, different analytical columns were tested. The main element for this research was arsenic, so the analysis was optimized in all conditions to separate the As(III), As(V), DMA, MMA, and AsB forms. After finding the optimal conditions and separating all of the arsenic forms, further elements were added, and the effectiveness of the analysis was checked. Out of the various types of columns, the following were tested:

- PRP-X100 (250 mm × 4.1 mm, 10 μm), packing material type: PS-DVB/Trimethyl ammonium exchanger (Hamilton, Reno, NV, USA);
- PRPX-200 PEEK (250 mm × 4.6 mm, 10 µm), packing material type: PSDVB/Sulfonic Acid (Hamilton, USA);
- BioWAX (50 mm × 2.1 mm, 5 µm), packing material type: nonporous/dietyloamine (Agilent, Santa Clara, CA, USA);
- Supelco SAX (250 mm × 4.6 mm, 5 µm), packing material type: silica gel, spherical particle platform/propyltrimethylammonium phase (Sigma–Aldrich, USA);
- Waters SAX (250 mm × 4.6 mm, 5 µm), packing material type: silica-based quaternary ammonium bonded sorbent (Waters, Milford, MA, USA);
- Dionex CS5A (250 mm × 4 mm, 9 µm), packing material type: latex, DVB/Cation: Sulfonic Acid/Anion: Quaternary Ammonium (Thermo, Waltham, MA, USA);
- Dionex CG5A (50 mm × 4 mm, 9 µm), packing material type: latex, DVB/Cation: Sulfonic Acid/Anion: Quaternary Ammonium (Thermo, USA);
- Dionex AS22 (250 mm × 4 mm, 6 µm), packing material type: DVB/Alkanol Quaternary Ammonium Ion (Thermo, USA);
- Dionex AG22 (250 mm × 4 mm, 6 µm), packing material type: DVB/Alkanol Quaternary Ammonium Ion (Thermo, USA).

Based on the literature describing arsenic, antimony, chromium, and selenium speciation analysis, different mobile phases were chosen for the experiment. Of the various types of mobile phases, ammonium carbonate (NH_4_)_2_CO_3_, ammonium bicarbonate NH_4_HCO_3_, nitric acid HNO_3_, ammonium nitrate NH_4_NO_3_, and ethylenediamine tetraacetic acid EDTA were examined (Sigma–Aldrich, USA). The best resolution results were obtained for NH_4_NO_3_. The addition of methanol was also tested. Three concentrations of methanol (1%, 2%, and 3%) in the mobile phase were tested. The pH value was modified in a range from 3.0 to 9.0. It was possible to obtain most of particular forms of analyzed metals with a good resolution only with the application of the Dionex IonPac AS22 with the Dionex IonPac CG5A guard column. Moreover, the most satisfactory analytical signals were obtained for concentrations of 1% and 2% methanol, so for further analysis 1% methanol was chosen. Unfortunately, these conditions were not optimal for speciation analysis of all forms of the chosen elements. A particular problem was the pH of the mobile phase, which was the key to the selection of analytes for further analysis. None of the forms of chromium could be separated in any analytical combination. The antimony forms were separated only in an acidic pH. However, it was possible to separate all arsenic and selenium species, though this required the use of an alkaline pH. In connection with the above, it was decided to continue and further optimize the analysis of only the arsenic and selenium species. The best resolution of the selenium forms was obtained in an acidic pH of 3.0, but at such an acidic pH we could not obtain a signal from arsenobetaine, which required a pH of 9.0. It was this pH that was chosen for further analysis. The influence of the column type and mobile phase on the separation effect is presented in table (Table 2).

### 3.4. Oven Temperature Effect

The optimization procedure for arsenic and selenium analysis was also extended by the oven temperature effect. The oven temperature was set at 10, 20, 30, 40, and 50 °C and was checked regularly. The best resolution and separation of the analyzed forms of As and Se were established at 40 °C, and this temperature was chosen for further speciation analysis.

### 3.5. Final Method 

Summarizing all optimization steps, the chosen analytical columns, eluent composition, and its pH were suitable to separate efficiently speciation forms of arsenic and selenium with significantly short RTs and acceptable signal-to-noise ratio. Unfortunately, the optimized speciation was not appropriate for speciation of chromium and antimony forms. Below, the characterization of speciation method of arsenic and selenium is presented. An anion exchange column Dionex IonPac AS22 analytical column (Thermo, USA) containing an alkanol quaternary ammonium ion and a cation–anion exchange analytical column and a Dionex IonPac CG5A analytical guard column (Thermo, USA) containing a fully sulfonated latex for cation exchange as the first layer and a fully aminated layer for anion exchange as the second layer were used. The total analysis time was set to 12 min. The mobile phase gradient elution was set to 0–2 min 100% mobile phase A, 2–4 min 100 → 0% mobile phase A and 0 → 100% mobile phase B, 4 min–10 min 100% mobile phase B, and 10–12 min 100% A. The mobile phases were aqueous solutions of 1b mmol·L^−1^ NH_4_NO_3_ (mobile phase A) and 75 mmol·L^−1^ NH_4_NO_3_ (mobile phase B), both at pH 9.0, adjusted by NH_4_OH. Methanol (1%) was added to the mobile phases to enhance the signal response for arsenic [31]. The oven temperature was set to 40 °C with a flow rate of 1.0 mL min^−1^, and the injection volume was set to 100 μL. All analytes were quantified on the basis of external calibrations constructed using standards prepared on the day of the analysis. Calibration curves for the As species (AsB, As(III), DMA, MMA, and As(V)) and Se species (Se-Met, Se-cystine, Se(IV), and Se(VI)) were constructed at concentrations of 0.1, 0.25, 1.0, 5, 10, 25, and 100 μg·L^−1^. Analytes as speciation forms of arsenic and selenium were identified by matching their retention times with those of standards. Using optimized chromatographic conditions, the retention times for all arsenic species were in the range from 2.4 min to 9.7 min, and the order of elution was AsB, As(III), DMA, MMA, and As(V). The retention times for all selenium species were in the range from 2.7 min to 10.1 min, and the order of elution was Se-Met, Se-cystine, Se(IV), and Se(VI). The accuracy of new speciation method was evaluated by analysis of the certified reference material Tuna Fish Tissue BCR-627. It is worth mentioning that there is a lack of Certified Reference Materials (CRMs), which are able to simultaneously determine all the forms of arsenic, which were separated by new speciation method. The main aim was to compare the arsenic speciation forms AsB and DMA. The obtained recovery for dimethylarsinic acid was 97 ± 5% and 98 ± 3% for arsenobetaine. The obtained results confirmed that nothing is held in the analytical column, suggesting that both the separation and detection elements of the analytical system were working correctly. The characteristics parameters of new method are presented in Table 3.

### 3.6. Samples and Sample Preparation 

The samples were collected from several convenience shops in urban areas in Poznan (Poland). Red shrimps, clams, octopus, squids, Argentine red shrimps, white blanched shrimps Vannamei, white shrimps Vannamei, bio white onion, pink onion, sweet white onion, and bio red onion were chosen for analysis. Tuna Fish Tissue BCR-627, Institute for Reference Materials and Measurements (IRMM), Geel, Belgium, with certified contents of arsenobetaine and dimethylarsinic acid, was used. The certified values were arsenobetaine, 52 ± 3 µmol·kg^−1^, and dimethylarsinic acid, 2.0 ± 0.3 µmol·kg^−1^ [56]. All food samples were first frozen and lyophilized for 48 h in a freeze dryer GT2E type 11 with a vacuum pump Leybold Trivac D5E. The mass 1.00 ± 0.01 g of the dried samples, which included red shrimps, clams, octopus, squids, Argentine red shrimps, white blanched shrimps, Vannamei white shrimps, bio white onion, pink onion, sweet white onion, and bio red onion, were weighed precisely in plastic tubes. The extraction process was carried out with 10 mL of distilled ultrapure water obtained from a Milli-Q Direct 8 purification unit (Millipore, Burlington, MA, USA, Merck) at 40 °C. Times of 20, 40, and 60 min were chosen to check the effectiveness of the water extraction process. The concentration of each determined species was constant, and because of that, the time of 20 min was chosen for further analysis. The water extracts of samples were cooled to room temperature and centrifuged (5.0 min, 14,500 rpm, MiniSpin plus, Eppendorf, Warszawa, Poland). Supernatants (water-soluble extracts) were carefully decanted, filtered through a 0.45 μm pore sized polyvinylidene difluoride syringe filter (Whatman, GE Healthcare Life Sciences, Piscataway, NJ, USA), and introduced directly to the HPLC–ICP–MS system. The samples (3 replicates) of CRM were prepared and analyzed with the same procedure as all the food samples. Moreover, portions of the foods and CRM extracts were analyzed by ICP–MS to determine the water-soluble concentration of analyzed metals. The parameters of the ICP–MS spectrometer for analysis are presented in Table 4.

## 4. Conclusions

The method presented here allowed for simultaneous speciation analysis of five analytical signals of arsenic forms, As(III), As(V), DMA, MMA, and AsB, and four analytical signals for selenium forms, Se(IV), Se(VI), Se-Met, and Se-cystine. Separation of the main forms of As, Cr, Sb, and Se was not possible during one single analytical run on one analytical column and mobile phase. The proposed method showed good selectivity, linearity, and sensitivity for arsenic and selenium species with the use of two analytical columns, Dionex IonPac AS22 + Dionex Ion Pac CG5A, and ammonium nitrate at pH = 9.0 as a mobile phase. The presented method was able to provide the necessary data for arsenic and selenium species condensed into one chromatogram. The new method developed allowed for the simultaneous determination of nine speciation forms, and among the analyzed onion samples, three forms were determined for one sample (white bio onion), and four for two seafood samples (red shrimp and squid). The iAs was obtained only in all onion samples (form As(III)), and the dominant form of organic As species in all seafood was AsB. In the case of all selenium, all forms were separated but for the one sample. The domination of SeMet forms was observed in all samples, especially in onions. The data obtained in water extracts on arsenic and selenium by ICP–MS analysis were comparable with the results from speciation analysis. This new method can be successfully applied for the speciation analysis of samples with similar natures and matrices.

## Figures and Tables

**Table 1:** Concentrations in ng·g^−1^ (except AsB which is in µg·g^−1^ *) of Water-Soluble Arsenic Species along with As in water-soluble results from ICP–MS in µg g^−1^, and Chromatographic Recovery Values (Limit of Detection (LOD’s) in µg·g^−1^ are AsB-16.59; AsIII-12.58; AsV-10.27; MMA-2.29; DMA-11.33; SeIV-3.59; SeVI-11.16; Se-cystine-23.07; Se-methionine-19.87).

Sample/Determined Form	As(III)	As(V)	MMA	DMA	As-Bet *	Water-Soluble as by ICP–MS	Column Recovery *
bio white onion	166.1 ± 7.5	<LOD	<LOD	<LOD	<LOD	0.171 ± 0.002	97.7 ± 3.7
pink onion	139.2 ± 5.6	<LOD	<LOD	<LOD	<LOD	0.153 ± 0.003	92.8 ± 4.7
sweet onion	149.3 ± 5.9	<LOD	<LOD	<LOD	<LOD	0.164 ± 0.002	93.3 ± 4.8
bio red onion	189.3 ± 7.9	<LOD	<LOD	<LOD	<LOD	0.206 ± 0.005	94.6 ± 4.7
red shrimp	<LOD	<LOD	<LOD	<LOD	15.48 ± 0.62	16.50 ± 0.09	93.9 ± 3.9
clam	<LOD	<LOD	336.9 ± 12.4	<LOD	13.32 ± 0.58	13.91 ± 0.17	98.2 ± 4.8
octopus	<LOD	<LOD	<LOD	<LOD	2.419 ± 0.112	2.511 ± 0.032	96.4 ± 3.8
squid	<LOD	<LOD	<LOD	<LOD	4.691 ± 0.221	5.011 ± 0.061	93.7 ± 4.4
red big shrimp	<LOD	<LOD	<LOD	119.8 ± 5.5	20.69 ± 0.782	21.30 ± 0.51	97.7 ± 3.7
white blanched shrimp Vannamei	<LOD	<LOD	<LOD	<LOD	2.507 ± 0.100	2.652 ± 0.032	94.6 ± 2.7
white shrimp Vannamei	<LOD	<LOD	<LOD	<LOD	2.221 ± 0.931	2.304 ± 0.027	96.6 ± 4.8
sample/determined form	Se(IV)	Se(VI)	Se-Met	Se-cystine	water-soluble Se by ICP–MS	Column recovery *	
bio white onion	<LOD	9.101 ± 0.302	609.7 ± 12.1	<LOD	0.671 ± 0.009	92.36 ± 4.41	
pink onion	959.2 ± 45.8	<LOD	<LOD	<LOD	0.994 ± 0.021	96.89 ± 4.75	
sweet onion	<LOD	<LOD	8788 ± 431.5	<LOD	8.921 ± 0.107	98.74 ± 3.85	
bio red onion	<LOD	<LOD	5065 ± 217.8	<LOD	5.302 ± 0.153	95.57 ± 4.31	
red shrimp	<LOD	47.53 ± 1.91	417.1 ± 16.9	987.2 ± 37.4	1.611 ± 0.032	90.17 ± 5.23	
clam	<LOD	182.6 ± 7.6	<LOD	<LOD	0.203 ± 0.003	91.32 ± 4.75	
octopus	<LOD	19.21 ± 0.92	435.5 ± 17.4	<LOD	0.502 ± 0.009	91.01 ± 4.55	
squid	<LOD	67.41 ± 2.73	<LOD	202.2 ± 8.1	0.287 ± 0.006	96.31 ± 4.29	
red big shrimp	<LOD	137.6 ± 5.7	<LOD	<LOD	0.152 ± 0.003	91.74 ± 3.89	
white blanched shrimp Vannamei	94.5 ± 4.6	<LOD	760.3 ± 32.3	<LOD	0.879 ± 0.017	97.15 ± 3.79	
white shrimp Vannamei	<LOD	<LOD	776.9 ± 36.4	<LOD	0.854 ± 0.011	91.41 ± 4.29	

* Chromatographic recovery (%) = (sum of the concentrations of water-soluble arsenic/selenium species/water-soluble arsenic/selenium by ICP–MS) × 100.

**Table 2:** The influence of the column type and mobile phase on the separation effect.

Column	Mobile Phase	As-Bet	As (III)	DMA	MMA	As (V)	Cr (III)	Cr (VI)	Se-Cystine	Se-Met	Se (IV)	Se (VI)	Sb (III)	Sb (V)
PRPX-100	HNO_3_ + CH_3_OH	✕	✕	✕	✕	✕	✕	✕	✕	✕	✕	✕	✕	✕
PRPX-200	HNO_3_ + CH_3_OH	✕	✕	✕	✕	✕	✕	✕	✕	✕	✕	✕	✕	✕
	NH_4_NO_3_ + CH_3_OH	✕	✓	✓	✕	✕	✕	✕	✕	✕	✕	✕	✕	✕
	NH_4_NO_3_ + CH_3_OH + EDTA	✕	✕	✕	✕	✕	✕	✕	✕	✕	✕	✕	✕	✕
BIOWAX	NH_4_NO_3_ + CH_3_OH	✕	✕	✕	✕	✕	✕	✕	✕	✕	✕	✕	✕	✕
SUPELCO	NH_4_NO_3_ + CH_3_OH	✕	✓	✓	✕	✕	✕	✕	✕	✕	✕	✕	✕	✕
WATERS	NH_4_NO_3_ + CH_3_OH	✕	✓	✓	✕	✕	✕	✕	✕	✕	✕	✕	✕	✕
DIONEX CG5A	NH_4_NO_3_ + CH_3_OH	✕	✕	✕	✕	✕	✕	✕	✕	✕	✓	✓	✕	✕
DIONEX CS5A	NH_4_NO_3_ + CH_3_OH	✕	✕	✓	✓	✓	✕	✕	✕	✕	✕	✕	✕	✕
DIONEX AG22 + AS22	NH_4_NO_3_ + CH_3_OH	✕	✕	✓	✓	✓	✕	✕	✓	✓	✓	✓	✕	✕
DIONEX AS22 + CG5A	NH_4_NO_3_ + CH_3_OH alkaline pH	✓	✓	✓	✓	✓	✕	✕	✓	✓	✓	✓	✕	✕
	NH_4_NO_3_ + CH_3_OH acidic pH	✕	✕	✕	✓	✓	✕	✕	✓	✓	✓	✓	✕	✕
	(NH_4_)_2_CO_3_ alkaline pH	✕	✓	✓	✓	✓	✕	✕	✕	✕	✓	✓	✕	✕
	NH_4_NO_3_ + NH_4_HCO_3_	✕	✕	✓	✓	✓	✕	✕	✓	✓	✓	✓	✕	✕
	NH_4_HCO_3_alkaline pH	✕	✓	✓	✓	✓	✕	✕	✓	✓	✓	✓	✕	✕
	(NH_4_)_2_CO_3_ + NH_4_HCO_3_	✕	✕	✓	✓	✓	✕	✕	✓	✓	✓	✓	✕	✕

✕, not separated form; ✓, separated form.

**Table 3:** LC–ICP–MS method characteristic.

	As(III)	As(V)	MMA	DMA	AsB	Se(IV)	Se(VI)	SeMet	Secystine
Conc. Range	Up to 100 µg·L^−1^ *								
Slope	9.004	21.08	8.863	0.495	7.749	3.407	6.405	4.087	4.086
R^2^	0.9999	0.9999	0.9999	0.9995	0.9993	0.9999	0.9999	0.9997	0.9993
Limit of detection	1.257	1.027	0.229	1.133	1.659	0.359	1.116	1.987	2.307

* Depends on loop volume (data for loop volume: 100 µL)—possible injection volume range: 10–500 µL.

**Table 4:** ICP–MS parameters for As and Se analysis.

Generator power [W]	1200
Argon flow—plasma [L/min]	8.0
Argon flow—nebulizer [L/min]	1.1
Argon flow—auxiliary [L/min]	0.7
Nebulizer	Concentric type “micro”
Torch	Concentric type “mini”
Spray chamber temperature [°C]	5.0
Collision gas—He [mL/min]	6.0
Voltage on octapole rods [V]	−21
Energy filter [V]	7.0
Sampling deep [mm]	5.0

## Data Availability

Not applicable.
